# DDT in Malaria Control: Roberts and Tren Respond

**DOI:** 10.1289/ehp.1002279R

**Published:** 2010-07

**Authors:** Donald Roberts, Richard Tren

**Affiliations:** Uniformed Services University of the Health Sciences, Bethesda, Maryland, E-mail: droberts@usuhs.mil; Africa Fighting Malaria, Washington, DC

Herren and Mbogo’s critique of our response (Tren and Roberts 2010) to van den Berg (2009) is lacking in substance. In their letter, they attack our work by characterizing our advocacy for using DDT to control malaria as a distraction from larger malaria control issues. These authors apparently discount the fact that some African countries are presently making highly effective use of DDT to reduce both malaria deaths and malaria infections. Countries that use DDT benefit from its spatial repellent action that stops mosquitoes from entering houses and transmitting disease, and no alternative insecticide does this (Roberts and Tren 2010). In addition, Herren and Mbogo apparently do not understand that our advocacy is consistent with that exhibited by the malaria control community, with hundreds signing a petition to prevent DDT elimination through Stockholm Convention negotiations. If DDT had been eliminated, countries presently using DDT would have been deprived of its benefits for protecting health and saving lives. Herren and Mbogo claim that our response to van den Berg’s commentary (van den Berg 2009) was fixated on DDT, in lieu of addressing the larger issues of what should be done to control malaria. In our letter (Roberts and Tren 2010), we addressed what we considered to be an attack on DDT use. How could we have responded without addressing the issues in van den Berg’s commentary?

Herren and Mbogo mischaracterize our position vis-à-vis DDT and alternative insecticides by asserting that we are reducing the malaria control debate to a simplistic equation of malaria or DDT. In fact, we have a public record of supporting the use of insecticide-treated nets and the use of alternative insecticides for malaria control. However, we have repeatedly emphasized that, for obvious reasons, insecticide-treated nets are not the only solution for malaria control. In fact, we object to a theme of nets and nets alone as much as we would object to a theme of DDT and DDT alone. Basically, there is no single-solution approach to malaria control. All tools are needed—not just those that are currently in vogue.

Herren and Mbogo state that they are fully aware that malaria is a worse outcome than possible health effects of DDT. We agree with them and appreciate their willingness to admit this, because their admission opposes published speculations that DDT might be causing more harm than good (Chen and Rogan 2003).

Herren and Mbogo conclude that we “do more to fuel those ‘interminable debates’ [DDT or no DDT for malaria control] than to meaningfully inform decisions that will save people’s lives.” It seems that these authors ignore the fundamental fact that we do not elaborate on alternative approaches to malaria control because the alternatives are not presently under threat of elimination. The alternatives are being used and should continue to be used, but the future is far less certain for DDT. Advocacy saved DDT from being eliminated during the original negotiations for the Stockholm Convention, and lives are being saved and diseases prevented as a consequence. The idea that the threat is over and that DDT is now available to those countries making effective use of it is wrong. The Stockholm Convention Secretariat is now planning to stop all production of DDT in 2017 and eliminate it entirely from use in malaria control programs in 2020 (UN Environment Program 2010). The Stockholm Convention Secretariat plans to prevent future uses of DDT, even though there is no cost-effective replacement for DDT. Given these circumstances, Herren and Mbogo should expect the interminable debates to become even more polemic in the future.

As for the big issues of what should be done to control malaria, our position is clear: Decisions should be based on scientific evidence of what actually works, on local circumstances, and on what proves to be the most cost-effective in terms of reducing disease and preventing human deaths.
